# Evaluating the user experience of the Beacon Decision Support System: a mixed-methods study of a digital platform for equity-oriented cancer care

**DOI:** 10.1186/s12913-026-15210-w

**Published:** 2026-07-27

**Authors:** Margherita Dahò, Dario Monzani, Veronica Coppini, Maria Vittoria Ferrari, Giulia Ferraris, Roberto Grasso, Victor Galvão, Chloe Laurent, Aline Machiavelli, Ricardo Pietrobon, Lucas Teixeira, Gabriella Pravettoni

**Affiliations:** 1 Department of Psychology, Educational Science and Human Movement (SPPEFF), University of Palermo, Viale delle Scienze ed. 15, 90128 Palermo, Italy; 2 Applied Research Division for Cognitive and Psychological Science, IEO, European Institute of Oncology IRCCS, Milan, Italy; 3 Department of Oncology and Hemato-Oncology, University of Milan, Milan, Italy; 4 SporeData OÜ Tallinn, Tallinn, Estonia

**Keywords:** Acceptability, Cancer, Decision support system, Digital health, Human-computer interaction, General psychology, Medical disparities, Usability, User experience

## Abstract

The Beacon Decision Support System (DSS) is a digital platform developed under the EU-funded BEACON project to address disparities in cancer care across Europe. It aims to provide patients, clinicians, researchers, and policymakers with centralized, validated access to oncology information. To evaluate the usability and acceptability of the Beacon DSS, we used a complementary qualitative and quantitative evaluation consisting of two independent studies aimed at informing the iterative development of a patient-centered and stakeholder-relevant tool. Study 1 involved qualitative interviews with 10 participants (five cancer patients and five oncology professionals/researchers) to assess usability, barriers, and expectations. Study 2 used a quantitative survey administered to 45 stakeholders, combining the System Usability Scale (SUS) and AttrakDiff Mini to assess functional and experiential aspects of usability. Patients appreciated the DSS’s visual clarity but reported technical issues such as app freezing and language accessibility, which were promptly resolved and did not persist in subsequent use. Professionals valued resource credibility but noted limitations in search specificity and data coverage. SUS scores (M = 60.3, SD = 9.6) indicated acceptable usability. AttrakDiff results showed strong scores for pragmatic quality and overall attractiveness, but more modest scores for hedonic quality. Feedback led to enhancements, including better filtering, glossary links, language geolocation, and onboarding tutorials. The Beacon DSS is perceived as a useful and credible tool for improving oncology navigation. Addressing usability challenges, particularly in personalization, search logic, and content accessibility, may further enhance the platform’s usefulness and accessibility for a broad range of users.

**Supplementary Information:**

The online version contains supplementary material available at https://doi.org/10.1186/s12913-026-15210-w.

## Clinical trial number

Not applicable.

## Introduction

Cancer is the second leading cause of death across Europe [1]. Yet, outcomes and access to high-quality care remain deeply unequal across European Union (EU) member states. Up to 25% of five-year cancer survivors, for the same cancer stage, experience disparities in access to care, particularly disadvantaging patients in poorer and more rural areas across Europe [2–5]. These inequalities are fueled by multiple, intersecting factors such as lack of access to accredited or specialized cancer centers, low health literacy, systemic fragmentation of care, and insufficient support for patients navigating the complex oncology pathway [2–4]. Participation in clinical research also reflects these disparities [6–9]. Indeed, patients from under-resourced regions are significantly less likely to be enrolled in clinical trials, further exacerbating inequities in access to cutting-edge treatments and personalized therapeutic options [10]. Language barriers, logistical constraints, researchers’ bias, and the absence of transparent, accessible information about available trials may contribute to this underrepresentation [9, 11]. Moreover, individuals with psychiatric or neurological conditions, whether pre-existing or developed because of cancer diagnosis and treatment [12, 13], are frequently excluded from research participation [6, 8, 9]. These exclusions are often justified on the grounds of protocol compliance or safety concerns; however, the risk is that they further marginalize already vulnerable populations and skew the evidence base away from those who may benefit most from support and innovation.

In response to these persistent disparities, a range of digital interventions has been proposed to support different aspects of cancer care. For instance, digital tools designed to empower cancer patients through interactive features, self-management support, and enhanced patient activation have shown positive effects on outcomes such as self-efficacy and coping. However, evidence varies across studies and contexts [14]. These tools often provide symptom tracking, educational resources, or interactive engagement to support patients throughout their care journey [14]. Other digital health solutions focus on supporting communication between cancer patients and healthcare providers, facilitating symptom reporting, shared decision-making, and patient-centred interactions [15]. Additionally, digital care platforms have been evaluated for their role in enhancing continuity of care and patient-reported outcomes through the integration of patient information and self-efficacy support, which illustrates the potential for digital solutions to positively impact aspects of cancer care delivery [16].

### The Beacon project and Beacon Decision-making Support System

Within this evolving landscape of digital interventions aimed at improving access and equity in cancer care, the BEACON project (Bridging European Access to Cancer Oncology Networks), funded under the EU4Health program, was intended to support efforts in reducing these disparities [4, 17, 18]. A key innovation of the project is the Beacon Decision Support System (DSS). While early definitions of decision support systems often emphasized sophisticated analytical models or Artificial Intelligence (AI) [19–21], a clinical DSS is broadly defined in contemporary literature as any system that “improves healthcare delivery by enhancing medical decisions with targeted clinical knowledge, patient information, and other health information” [22]. According to this framework, a DSS does not necessarily rely on automated or prescriptive algorithms. Instead, it supports decision-making by organizing, integrating, and presenting structured knowledge that enables users to make informed choices [22]. Within this perspective, the Beacon platform can be considered a DSS as it aggregates and structures pan-European cancer-related information through an advanced ontology, allowing users to retrieve targeted information that informs decisions in specific clinical and organizational contexts.

More specifically, the platform supports decision-making for four key stakeholder groups:

- *Patients*: By helping them identify appropriate treatment centers, compare available healthcare services, and locate relevant clinical trials.
- *Clinicians*: By facilitating referral decisions and treatment planning through access to evidence-based guidelines, specialized centers for rare cancers, and information on ongoing clinical trials.
- *Researchers*: By enabling the identification of research priorities, appropriate data sources, and differences in service availability across European regions.
- *Policymakers*: By providing aggregated evidence on cancer disparities and healthcare system capacities to inform strategic planning and resource allocation.

Compared with existing digital interventions, the Beacon DSS is distinctive for its explicit focus on improving equity in access to cancer care through an integrative design approach. The platform combines pan-European data sources, multilingual support (24 languages), and tailored interfaces for multiple stakeholder groups, including patients, healthcare professionals, researchers, and policymakers. To detail how the system serves the diverse users, table S1 (see Supplementary File 1) summarizes the specific platform functionalities, intended benefits, and core use cases for each of the four stakeholder profiles.

**Fig. 1:**
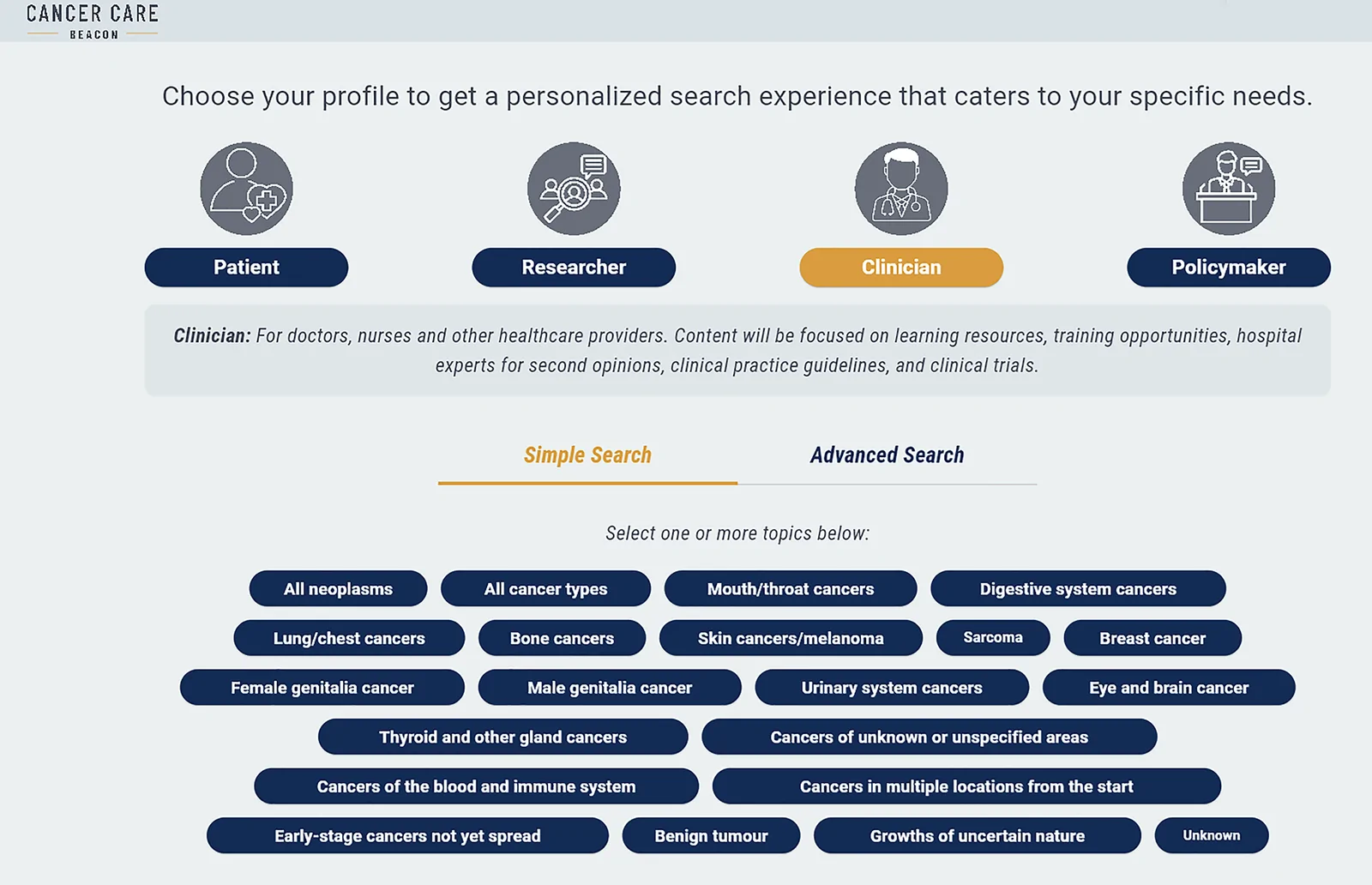
Homepage of the Beacon DSS. When a user selects a stakeholder profile (patient, clinician, researcher, or policymaker), the interface visually adapts by changing the color scheme and updating the displayed content to reflect information and resources tailored to that specific user group

By aggregating and validating information from diverse national and international sources into a single platform, the DSS aims to reduce informational complexity and navigational barriers across heterogeneous healthcare systems. Navigational barriers are particularly salient when care pathways, eligibility criteria, or reimbursement policies differ across regions or countries, making it difficult for patients to identify and access appropriate services [4, 17, 18]. Unlike the fragmented and often unverified information that users may encounter when searching across multiple online sources, the Beacon DSS aggregates and handles information from verified sources within a single platform. By providing centralized, trustworthy, and multilingual information, the DSS is intended to improve transparency, support informed decision-making, and facilitate timely identification of relevant healthcare services and resources. Furthermore, the multi-stakeholder, transnational approach addresses a gap in the literature on DSS designed to facilitate equitable access to oncology services. Overall, the Beacon DSS aims to promote equity by enhancing transparency, empowering users with trustworthy information, and facilitating a more informed alignment between healthcare resources and patients’ needs. Figure 1 shows the Beacon DSS homepage.

To ensure the system’s evidence base and functionality, a range of methods and data sources were used. Insights from a modified DELPHI survey and Natural Language Processing (NLP) analyses helped identify key policy-relevant themes, including the role of patient associations and the implementation of evidence-based practices. These inputs informed the creation of the Cancer Disparity Atlas, which visualizes differences in incidence and mortality across cancer types and European countries. The Atlas also integrates structured data from 82 hospitals (mostly OECI-accredited), 60 claims databases, 77 registries, and 15 additional datasets, including biobanks, surveys, and supplementary resources such as policy reports and patient associations. Furthermore, to enhance interoperability and advanced analytics, the DSS data are structured using the Resource Description Framework (RDF), with elements tagged via standard vocabularies such as Dublin Core. This allows the data to be transformed into RDF triplets, which can be queried using SPARQL and analyzed using machine learning or statistical methods. The structured approach enabled the DSS to deliver targeted insights for each stakeholder group, supporting tailored access for patients, clinicians, researchers, and policymakers. Experts’ insights further refined both resource-allocation algorithms and the system’s interface, ensuring that critical information, such as hospital capabilities, was clearly highlighted to support decision-making.

### Study design overview

The ongoing need to identify and understand non-measurable aspects of user experience with software has long motivated researchers in Human-Computer Interaction (HCI) [22–24]. Indeed, developing a digital health platform alone does not guarantee its success. The usability and acceptability of such systems, particularly in emotionally and cognitively complex domains like oncology, are essential to ensuring that users can and will adopt them and remain engaged over time [21–25]. Digital tools in cancer care must be intuitive, sensitive to the emotional context, and capable of delivering reliable information without adding to users’ burden. Therefore, this study aims to evaluate the user experience of the Beacon DSS through a complementary qualitative and quantitative evaluation based on two independent studies. This approach was designed to gain comprehensive insights into how different stakeholders interact with the system, perceive its usefulness, and identify potential barriers to use. The ultimate goal is to ensure that the Beacon DSS reflects the needs and preferences of its intended users and to inform evidence-based refinements that will maximize its effectiveness and real-world impact.

To explore how users interact with the Beacon DSS and to guide its iterative refinement, we conducted two studies. In the first, we conducted in-depth qualitative interviews with key stakeholders, including cancer patients and oncology professionals, to identify usability challenges, uncover unmet needs, and gather suggestions for system improvements. This study aimed to capture the lived experience of navigating the app, offering insights into both practical barriers and emotional responses. The second study expanded the evaluation to a broader group of users through a quantitative assessment. Participants completed two validated instruments - the System Usability Scale (SUS) [26] and the AttrakDiff Mini questionnaire [27] - to measure both functional usability and user experience.

By integrating qualitative and quantitative approaches in two independent yet complementary studies, this paper offers a comprehensive perspective on how diverse users perceive and interact with the Beacon DSS. The qualitative phase captures context-rich insights into user behaviors, expectations, and emotional responses, while the quantitative phase provides structured, measurable data to assess usability across a broader sample. Together, these complementary methods enable a multi-dimensional understanding of the system’s strengths and limitations and inform evidence-based refinements responsive to stakeholders’ needs. This research was approved by the Bioethical Committee of the University of Palermo (approval number n. 205/2024) and in accordance with the World Medical Association Declaration of Helsinki. All participants provided informed consent to take part in the study. The following sections describe in greater detail the specific procedures, instruments, and analytic strategies employed in each phase of the evaluation process.

## Methods study 1: Qualitative usability evaluation

### Participants and recruitment

A total of 16 individuals initially responded to the study invitation, including nine cancer patients and seven oncology professionals/researchers. After screening and scheduling, 10 participants were ultimately interviewed (five patients and five professionals).

#### Patient participants

Eligibility criteria for patient participants were: (a) age 18 years or older; (b) a current cancer diagnosis; (c) ability to speak Italian and understand and provide written informed consent; (d) basic technological literacy, defined as the ability to use web browsers for online information searching. Patients who did not meet the eligibility criteria and/or had stage IV cancer were excluded. Recruitment took place over one month through social media posts by the European Institute of Oncology (IEO). Of the nine patients who initially expressed availability, one declined due to lack of interest, one could not be scheduled, despite several attempts, and two withdrew their interview at the last minute due to health-related issues (chemotherapy side effects). Consequently, five explorative interviews were conducted.

The interviewed patients included three women and two men, aged from 42 to 68 years old (*M* = 55.4; *SD* = 10.7). Participants’ educational backgrounds included one degree in engineering, one in architecture, and two in psychology; one patient had a high school diploma. Diagnoses were breast cancer (*n* = 1), prostate cancer (*n* = 1), skin cancer (*n* = 1), head cancer (*n* = 1), and leukemia (*n* = 1). The small final sample reflects the challenges of recruiting and scheduling interviews with oncology patients, particularly those undergoing active treatment.

#### Oncology professionals and researchers

Of the seven professionals who initially responded, no specific inclusion or exclusion criteria were applied beyond their willingness to participate and relevant oncology expertise. Two professionals withdrew because it was impossible to arrange the interview, given their extremely busy schedules; consequently, five oncology professionals were interviewed. This group included two men and three women, aged 38–55 years (*M* = 47.3; *SD* = 5.6). Their specialties included breast oncology (*n* = 1), hematologic oncology (*n* = 1), gynecologic oncology (*n* = 1), neuro-oncology (*n* = 1), and surgical oncology (*n* = 1). Their clinical and research expertise provided complementary perspectives on patient experiences.

#### Policy makers

Initially, the study design aimed to include policymakers to capture a broader perspective on oncology care. However, despite several months of targeted recruitment, no policymakers were able to participate, primarily due to competing professional commitments and very limited availability. In this context, policymakers are often also hospital managers, and oncology policy positions are relatively scarce in the region, making them difficult to reach. Nevertheless, policymakers were successfully engaged in the complementary quantitative study (Study 2), allowing the collection of structured data on relevant system-level aspects. As a result, their absence from the qualitative phase does not compromise the study’s validity, which remains grounded in the lived experiences of the principal stakeholders.

### Procedure

Qualitative data were collected through one-on-one online interviews conducted by two researchers: a licensed clinical psychologist and postdoctoral researcher (MD), and an associate professor of general psychology (DM). Both interviewers possess relevant expertise in qualitative methods and usability evaluation. Participants recruited were randomly allocated to one of the two interviewers. All interviews were conducted in Italian and remotely on Zoom, lasted 50 to 60 min, and were audio recorded. Notes were also taken during the interviews to capture key observations and contextual details. The interview guide was developed based on established literature in usability research [28–30] and employed a predefined structure to ensure consistency by outlining the main areas of interest and essential questions in advance [31], ensuring comprehensive coverage of key app-related topics.

To ensure meaningful, informed feedback, each participant was asked to use the tool for about 20–30 min before their interview, though they were free to spend more time if needed. No formal training or instruction was provided, as the DSS is designed to function like a standard website, and all participants were selected to have at least basic digital literacy. Providing structured training could also have introduced bias, particularly regarding usability, whereas independent exploration allowed participants to directly assess the platform’s intuitiveness and ease of use. Despite the emphasis on autonomous exploration, participants were provided with a carefully designed list of suggested, generic activities to ensure that all key aspects of the DSS were explored. These included searching for information relevant to *their* specific cancer type, locating local hospitals or clinics, and exploring other core platform functions such as navigation across different content sections, language settings, and user account features. Participants were also encouraged to freely explore the platform and interact with the resources most relevant to their individual needs and interests. Together, these activities covered the principal functions of the DSS that users would typically access during routine use. This approach ensured that feedback was both structured and authentic, capturing meaningful insights into usability and content without introducing bias. Medical professionals tested the DSS from both clinical and research perspectives, navigating under different user profiles to observe how results varied depending on the selected role on the homepage, while following the same exploratory approach as the patients. Specifically, they were asked to explore the system using professional-specific filters to search for clinical data, guidelines, and trials relevant to their field of expertise. Similarly to patients, they were also encouraged to explore the platform freely in order to mimic naturalistic use and provide feedback on the functionalities most relevant to their needs.

Overall, all participants were adequately informed about the project and their task, ensuring that their interactions were meaningful, reflective, and conducted with full autonomy. This approach provided valuable insights into the app’s clinical utility, its potential integration into daily routine or patient care workflows, and its applicability as a research tool. It also enabled stakeholders to reflect on the platform’s strengths and limitations in relation to their real-world decision-making needs, while allowing them to explore features, familiarize themselves with the interface, and simulate tasks relevant to their roles.

During the interviews, participants were then invited to share their impressions and experiences and encouraged to provide comprehensive feedback covering multiple aspects of the tool, including usability, accessibility, design appeal, and content relevance. They were also prompted to suggest improvements or additional features to enhance the app’s effectiveness and better meet their needs. Table 1 summarizes themes and questions that guided the interviews. Importantly, while a shared framework was applied across interviews, the questions were tailored specifically to each stakeholder group to ensure relevance to their experiences, roles, and perspectives.

**Table 1:** Specific themes and guiding questions for the qualitative interviews

Themes	Questions
*1. Quantify the Potential Use of the System*	• Do participants envision using the DSS in their search routines? • In what scenarios could the DSS be most beneficial?
*2. Evaluate Usability and User Experience*	• How easy is it to navigate and complete tasks within the DSS? • What usability barriers did participants encounter?
*3. Assess Feasibility*	• How practical is it to integrate the tool into the existing daily life (for patients), or healthcare workflows (for specialists)? • Have participants found any technological barriers to implementation?
*4. Examine Accessibility*	• Is the tool compatible with various devices, operating systems, or browsers? (Participants were asked whether they had tried the DSS on different devices. Overall, a collective response was obtained, as each stakeholder tested the platform on their own device). • What accessibility challenges did stakeholders face?
*5. Evaluate Design Attractiveness*	• What are participants’ impressions of the visual design (colors, typography, aesthetics, icons)? • Which design features do users prefer for engagement and satisfaction?
*6. Solicit Suggestions for Improvement*	• What improvements to functionality or interface do participants suggest? • What additional features could better meet users’ needs?
*7. Assess Content Relevance*	• Is the information clear and appropriate? • Does the content align with users’ expectations and needs? • What extra functionalities (e.g., reminders, scheduling) are desired?

### Data analysis

Data coding and thematic development followed the principles of Framework Analysis [32, 33], a structured approach widely used in applied qualitative research, particularly in health and policy contexts. The analysis proceeded through the following stages. First, familiarity was established by repeatedly reading all interview transcripts to gain an overall understanding of the data. Second, an initial thematic framework was developed based on both the study aims and recurring issues identified in the data. Third, indexing was performed by systematically applying the thematic framework to the transcripts through manual coding. Fourth, charting involved summarizing coded data into thematic matrices to allow comparison across participants and stakeholder groups. Finally, mapping and interpretation were conducted to identify cross-cutting patterns, relationships between themes, and higher-order insights relevant to evaluating and refining the Beacon DSS. This method allows for both predefined objectives and emergent insights to be integrated into the analysis.

In this study, the analysis focused on three major themes: Usability Experience, Positive Aspects, and Suggestions for Improvement. While multiple frameworks exist to guide understanding of technology use, these three themes were selected to capture the most relevant and actionable aspects of user interaction with the DSS, ensuring the findings would directly inform its refinement. These themes also reflect the key domains of user interaction, most relevant to digital development and enhancement, and were developed iteratively through careful review of the interview transcripts, allowing the team to capture both anticipated issues and unexpected patterns in participants’ responses. Focusing on these three themes ensured that the analysis remained targeted, actionable, and aligned with the primary goals of improving usability and user satisfaction.

Coding was conducted independently by two authors (MD and DM), with subsequent verification and discussion by three additional scholars (VC, MVF, and GF) to enhance credibility, reduce bias, and discuss data saturation. To ensure consistency and reliability of coding, the coders first conducted a pilot assessment on a subset of transcripts, discussed discrepancies, and refined the coding scheme before coding the remaining data. Any disagreements during coding were resolved through discussion among the full research team, ensuring a consensual and credible thematic analysis. No qualitative data analysis software was employed; instead, manual coding and thematic categorization were performed. Participants did not provide feedback on the findings. Participant quotations are included in the results to illustrate key themes.

To ensure methodological rigor and transparency, the study adhered to the Consolidated Criteria for Reporting Qualitative Research (COREQ) checklist [34].

## Results study 1: Patients’ overview

The qualitative findings are organized around three higher-order analytic themes that emerged as central to users’ experiences with the DSS: perceived and enacted usability, experienced value and positive attributes, and user-identified directions for improvement. These themes represent cross-cutting patterns derived from an integrated analysis of the perspectives, capturing underlying determinants of how the DSS is perceived, used, and evaluated. Together, these themes move beyond surface-level description by identifying recurring mechanisms shaping user experience, which can inform the design and evaluation of similar oncology DSS. The themes are illustrated and substantiated in the following paragraphs.

### Usability experience

All participants reported encountering technical difficulties that modestly hindered their overall experience with the tool. A recurring problem was the freezing, especially when using the search functions, which interrupted their workflow and caused frustration. However, this issue was promptly resolved and did not persist in subsequent use. Additionally, the language selection process was not intuitive; the DSS did not automatically default to their preferred language, forcing users to adjust the settings, which some found confusing. Although login was optional, those who attempted to log in faced challenges that further complicated their use of the app. However, logging in would have granted access to additional features, such as saving search history and enabling a more personalized experience. Finally, slow loading times were another frequent issue that was promptly resolved. These delays made navigation feel cumbersome and detracted from the app’s usability. Together, these technical limitations initially affected the fluidity of the user experience but also provided valuable insights into key areas for refining the app’s performance and interface.

Example quote:

- “*I often had to wait quite a bit when searching for information*,* and sometimes the app would just freeze*,* which was frustrating*.” (Patient 2);
- *“The freezing is problematic*,* especially if I might be trying to quickly look something up.”* (Patient 4).

### Positive aspects

**Fig. 2:**
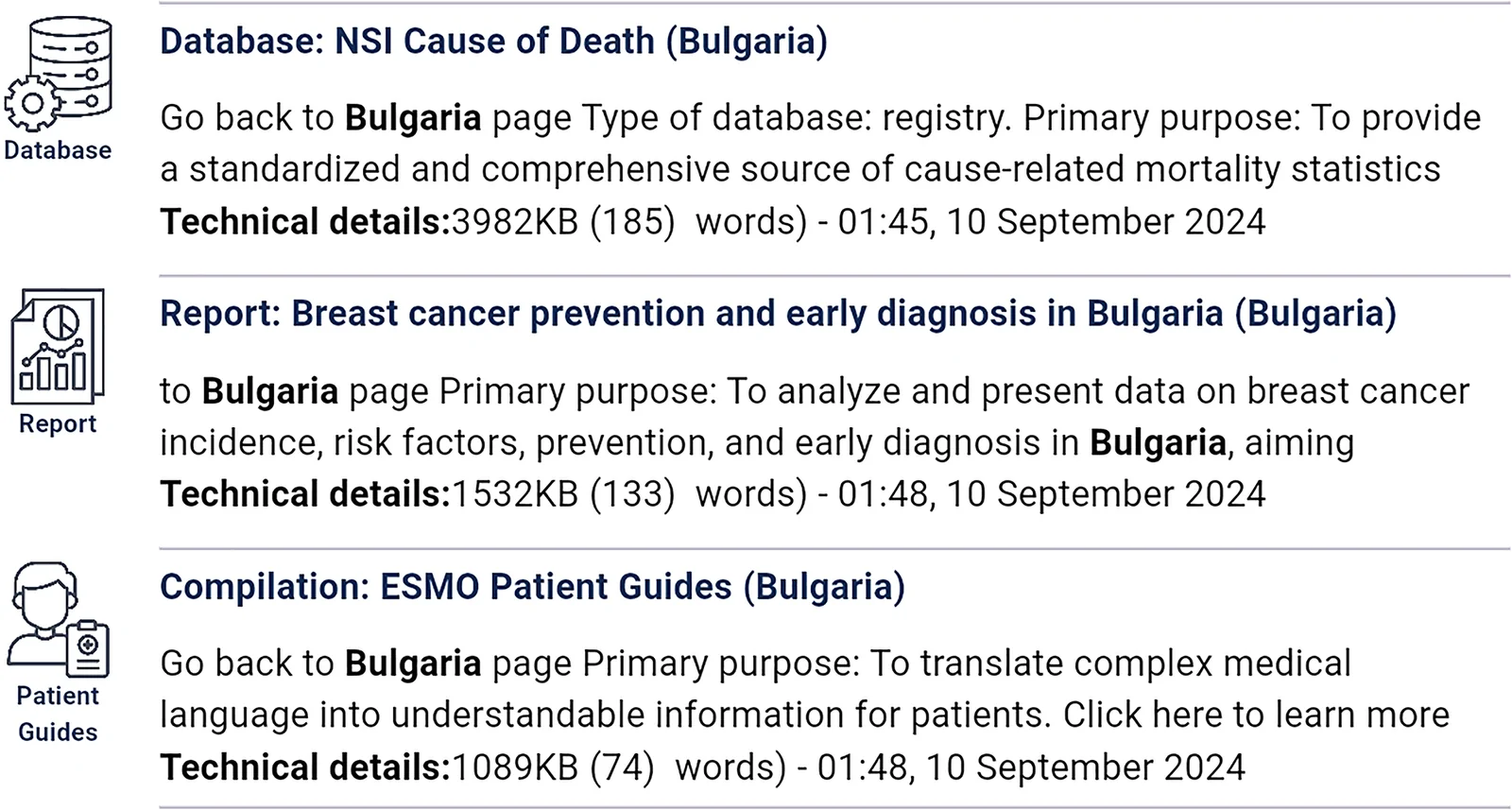
Example of a search result from the Beacon DSS. The results display a symbol and label for the type of resource, the title of the resource, the country of the resource, and a brief section based on the query search, as well as additional metadata

Despite the technical shortcomings, users generally appreciated the design and functionality once it was operational. They praised the clean and straightforward layout, which made navigation simple even for those less comfortable with digital technology. The visual design, including color choices and font sizes, was seen as accessible and readable, potentially suitable for older users or those with limited technical skills. The search filtering system was a standout feature (Fig. 2). Participants valued the ability to refine searches by criteria such as cancer type or treatment location, which helped prevent information overload and allowed them to quickly find relevant resources. Users also appreciated the detailed information available on specific cancer types and treatment centers, which they considered a strong asset. However, some noted that gaps in the database occasionally limited the information they could access.

Example quotes:

- *“The filters helped me narrow down what I was looking for without getting overwhelmed by too many options.”* (Patient 1);
- *“I liked how clear and simple the design was. It made using the app easy*,* even though I am not very tech-savvy.”* (Patient 4);
- *“The layout is well thought out. It is intuitive enough that I could recommend it to older patients without worrying they’d get lost.”* (Patient 3);
- *“From my side*,* the filtering by region and tumor type is very helpful”* (Patient 2).

### Suggestions for improvement

Participants provided several constructive recommendations to enhance the app, summarized in eight points: technical improvements, language settings, homepage organization, search display, accessibility features, terminology, database completeness, and patient-centered features.

1. *Technical Improvements*: As previously discussed, addressing issues such as app freezing and long loading times is critical. These problems were promptly reported to the technical team, who have been working to resolve them. However, one participant noted an improvement in performance during the interview itself:

*A couple of days ago*,* it kept freezing and was slow*,* but today*,* while I am using it during this interview*,* it’s already working much better.* (Patient 2);

2. *Language Settings*: The DSS should automatically open in the user’s local language, perhaps based on IP detection, and offer clearer options for manual language changes.

*It would be great if the app just opened in my language automatically*,* instead of me having to change it every time.* (Patient 5);

3. *Homepage Organization*: Participants suggested that introducing a more structured homepage with categorized menus (e.g., by cancer type, region, or treatment center) could simplify navigation beyond the current search-focused design.

*A menu where I can pick the type of tumor or location would help find what I need without typing so much.* (Patient 1);

4. *Search Results Display*: Users found the current results overwhelming. They suggested adding preset filters on the homepage to help refine searches early and clearer messages when no results are found.

*The results came all at once*,* and it was too much. Having filters from the start would make it easier to focus.* (Patient 3);

5. *Accessibility Features*: Enhancements such as adjustable text sizes and improved link visibility would improve readability and ease of use, as a patient noted:

*The font was fine for me*,* but older patients or those with visual difficulties might struggle - larger text options would be helpful.* (Patient 5);

6. *Database Completeness*: Expanding the database to cover rare or specific conditions more thoroughly would increase its usefulness.

*I tried to look up a rare subtype and got no results - it would be great if the database could include more detailed entries.* (Patient 3);

7. *Terminology and Language*: Participants recommended including a glossary for medical terms and using more patient-friendly language. Two participants specifically requested softer alternatives to the word “cancer,” such as “illness” or “condition,” to reduce emotional discomfort.

*Some of the words were really technical - I had to Google them. A glossary or simpler language would help a lot.* (Patient 4);

*Every time I see the word ‘cancer’ it hits me. I would prefer something like ‘condition’ - it feels softer.* (Patient 3);

8. *Patient-Centered Features*: Suggestions included adding interactive components like social networking for patients and more detailed clinical trial information.

*It would be nice if I could connect with other patients or at least see what trials are going on that I might be eligible for.* (Patient 2).

## Results study 1: Clinician-researcher’s overview

In line with the analysis of patients’ qualitative data, the findings from healthcare professionals are structured around the same three higher-order analytic themes, capturing cross-cutting patterns in user experience. These themes reflect the underlying determinants of how the DSS is perceived, navigated, and evaluated, and provide insights to refine the tool’s design and functionality.

### Usability experience

Clinicians generally perceived the Beacon DSS as a helpful tool for navigating oncology resources, though several usability limitations affected the overall experience. The most frequently cited concern was the lack of precision in the search function. Participants explained that entering specific queries - such as looking for clinical trials focused on rare cancers - often yielded overly broad or irrelevant results. Although filters could be applied after the initial search, this step was perceived as inefficient and time-consuming.

Example quote: *When I search for a rare cancer type*,* the results are too generic. I have to click through pages or reset filters constantly - it slows everything down.* (Clinician 2)

The current structure, which introduces filters only after a search, was considered counterintuitive. Many clinicians recommended that filtering options be available from the outset to improve workflow efficiency and avoid redundant navigation steps. In addition, language and translation issues emerged as a source of confusion. Some participants noted that key clinical terms appeared to have been automatically translated, leading to awkward or unfamiliar phrasing that disrupted comprehension - particularly in multilingual settings.

Example quote: *Some of the terminology feels like a literal translation. It does not match the medical vocabulary we use daily*,* which makes it harder to trust at first glance.* (Clinician 3)

A further point of concern was the incompleteness of the database. While major cancer centers were well represented, smaller OECI-affiliated hospitals had not yet contributed data, limiting the app’s coverage. Clinicians seeking information on sub-specialized services or lesser-known institutions found this gap problematic.

Example quote: *It would be useful to have more details from smaller centers. Not all patients go to the big institutes*,* especially in rural areas.* (Clinician 1)

### Positive aspects

**Fig. 3:**
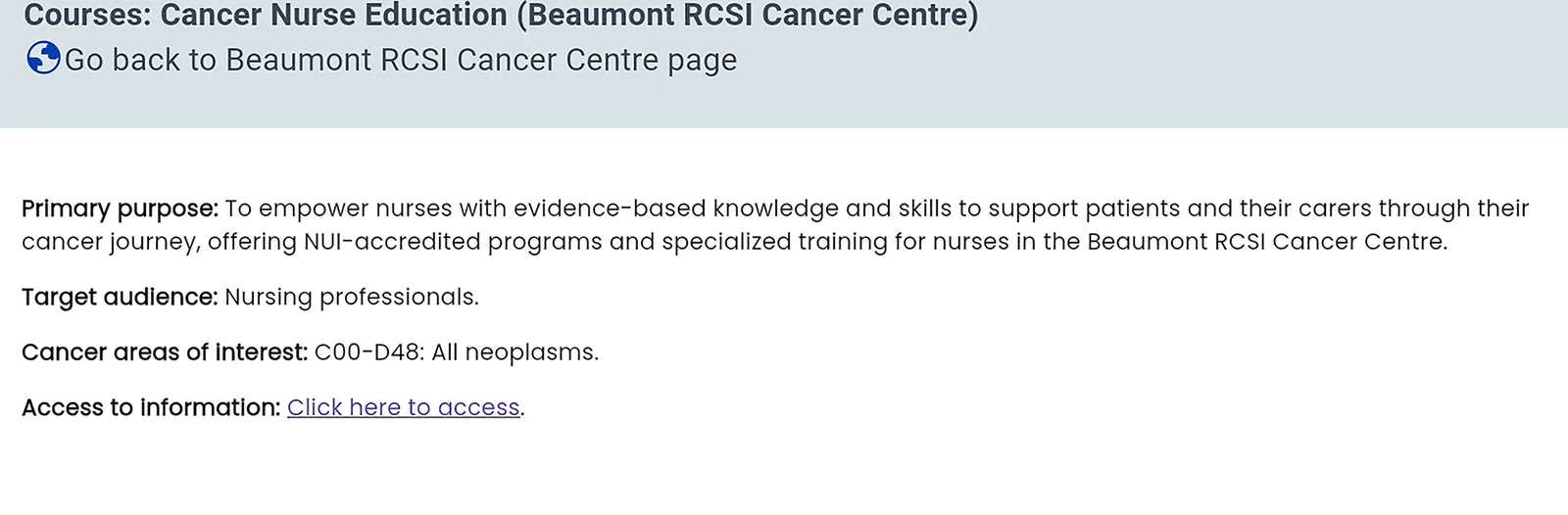
Example of a resource page in the Beacon DSS. Resource pages show the title of the resource, its primary purpose, the target audience, cancer areas of interest, a link to access the information, and other information based on the type of resource such as instructions to access databases

Despite the challenges reported above, clinicians highlighted several features that significantly enhanced their experience with the Beacon app. Chief among them was the reliability and credibility of the information provided. The app’s structured presentation of verified links (Fig. 3) to European cancer networks, clinical trials, and accredited institutions was seen as a valuable time-saving resource in clinical settings.

Example quote: *I appreciated having a single space where I could access high-quality resources without the need to cross-check everything across platforms.* (Clinician 5)

The interface design and visual layout were also praised for their clarity and professionalism. Clinicians found the DSS intuitive to navigate, with a clean aesthetic that supported focus and reduced cognitive overload. This was particularly helpful when using the app during time-sensitive situations, such as preparing a referral or discussing treatment options with patients. Moreover, several participants noted the app’s potential to facilitate professional networking and inter-institutional collaboration, thanks to its integration of directories and information on centers of excellence and training programs.

Example quote: *It is not just a search tool - it gives us access to centers*,* to expertise*,* and to each other. That’s incredibly valuable in oncology.* (Clinician 4)

### Suggestions for improvement

Clinicians provided a series of thoughtful, constructive recommendations to enhance the app’s clinical utility and user experience. Their suggestions focused on five main areas: refining the search system, broadening and standardizing content, improving accessibility, integrating AI-based support, and promoting wider awareness and training.

1. *Improving Search and Filtering Functions*: Clinicians stressed that enabling filters before a search would streamline the process. Currently, users must first perform a broad search and then apply filters, which might be inefficient. They suggested adding advanced filters on the homepage, including categories such as clinical specialties, resource types, and geographic regions. Options to narrow results by rare cancers or patient subgroups were also recommended to increase precision and usability.

Example quote: *The filter should come first*,* not after. It would save time and make searches more precise from the start.* (Clinician 1)

2. *Expanding and Standardizing Database Content*: Another key focus was the completeness and consistency of institutional profiles. Clinicians stressed the importance of including a wider range of hospitals, particularly smaller or regional OECI centers, to ensure equitable access to information. They suggested encouraging contributions from these less prominent institutions, standardizing the data shown for each center - covering diagnostic services, therapeutic options, and research activities - and adding information on palliative care, psychological support, and patient associations. These elements are vital for holistic oncology care but are often missing.

Example quote: *We need a comprehensive view of what each center offers*,* especially for referrals involving complex or rare conditions.* (Clinician 2)

3. *Enhancing Accessibility and Language Clarity*: Clinicians pointed to the need for a more user-friendly interface and clearer language, especially for non-expert users. They recommended a brief tutorial or onboarding guide to help users navigate the search system effectively, including Boolean operators and syntax. Simplifying technical jargon in patient-facing sections - particularly those about clinical trials and procedures - was also seen as crucial to prevent confusion or alienation.

Example quote: *Sometimes the language may be too technical for patients. Making it simpler would help them understand and feel more comfortable using the app.* (Clinician 4)

4. *Integrating AI and Patient Support Tools*: Looking ahead, clinicians saw great promise in AI-powered features to assist both professionals and patients. They proposed developing a virtual assistant to answer FAQs and guide users through the app, and to include qualitative insights into patient experiences and quality-of-life outcomes after treatment to foster shared decision-making.

Example quote: *An AI assistant could really help both clinicians and patients find quick answers*,* making the app feel more interactive and supportive.* (Clinician 3)

*5. Promoting Training and Awareness*: Finally, clinicians emphasized the importance of users understanding the app’s full potential. They suggested brief training sessions or digital toolkits for both clinicians and patients to improve confidence in using the app. Raising awareness across oncology networks would encourage wider adoption and more data sharing.

Example quote: *There’s a lot of potential*,* but some functions are hidden. A short tutorial could make a big difference.* (Clinician 5)

## Methodology study 2: Quantitative usability evaluation

### Participants recruitment

Participants attended the BEACON final conference event, held both in person (Milan, Italy) and online on 22nd–23rd October 2024. The free event was structured as a standard conference, open to all interested stakeholders, including patients, researchers, oncology professionals, policy makers, and patient association representatives. The first day focused on discussions with invited speakers around oncology disparities, digital health, and potential solutions, including a brief presentation of the Beacon-DSS to provide participants with context on the tool and its objectives.

The second day included a one-hour practical workshop focused specifically on the Beacon DSS. Participants who chose to take part were invited to interact with the tool in real time, following the same approach used during the interviews. After a deeper introduction to the system, participants were given approximately 30 min of hands-on interaction. To ensure a minimum level of comparability across participants while preserving autonomy, they were provided with a list of suggested, non-prescriptive tasks designed to prompt exploration of key system functionalities. The suggested activities were broadly aligned with those used during the qualitative evaluation and included searching for oncology-related information, exploring healthcare providers and services, applying filters, selecting preferred language, and navigating stakeholder-relevant content. Participants were free to deviate from these suggestions, allowing the observation of spontaneous use patterns and individual exploration strategies. During this period, participants could ask questions and discuss their observations live with the facilitator (AM from the technical team), allowing for immediate clarification and feedback.

After the workshop, and to further increase public engagement, usability surveys were completed step by step during the live session using Wooclap (https://www.wooclap.com), with aggregated results displayed immediately on the screen (session conducted by DM). Participation in the DSS evaluation was voluntary, and all participants provided informed consent. Given the open nature of the event, the sampling strategy was not restrictive. Based on real-time observation during the workshop session, survey completion appeared high, with only a small number of participants (primarily organizers and project staff) not completing the questionnaire. As the survey was administered in an open, live setting, a formal response rate could not be calculated. Furthermore, although participant backgrounds varied, this approach was considered appropriate for a quantitative assessment aimed at gathering diverse and meaningful feedback.

### Measures and data analyses

Alongside basic sociodemographic questions, two validated tools were employed to assess participants’ perceptions of the application. The first was the System Usability Scale (SUS) [26], a widely used and validated instrument for evaluating perceived usability. Items are rated on a five-point Likert scale ranging from *Strongly Disagree* to *Strongly Agree*.

The second was the AttrakDiff Mini [27], which uses ten pairs of bipolar adjectives to evaluate four dimensions of user experience: Pragmatic Quality (PQ), Hedonic Quality – Stimulation (HQS), Hedonic Quality – Identification (HQI), and Attractiveness (ATT). In addition to these standardized measures, participants were asked to provide an overall satisfaction rating on a 5-point scale. The two evaluation tools were selected because they capture complementary aspects of interaction with digital health tools. While the first instrument focuses primarily on usability and perceived ease of use, the AttrakDiff Mini allows for a broader assessment of user experience, capturing dimensions that are particularly relevant for DSS intended for diverse stakeholders. In prior research, both instruments have demonstrated good to excellent internal consistency, with Cronbach’s alpha values typically reported around 0.52-0.84 for the Italian SUS’ validation [35] and between approximately 0.85 for the AttrakDiff Mini dimensions [27].

Finally, an additional item assessing overall satisfaction with the app was included for descriptive purposes but was not incorporated into the SUS total score. Items are rated on a 5-likert scale ranging from *Not Satisfied* to *Extremely Satisfied.*

While the qualitative study provided in-depth insights into users’ experiences, the quantitative phase aimed to complement and triangulate these insights using standardized indicators; data were analyzed in IBM SPSS (Version 26.0), which was used to compute descriptive statistics and visualize results across the usability dimensions.

## Results study 2

### Participants’ demographics

A total of 45 participants participated in this experience. The group had a mean age of 38.3 years and was predominantly female (67%). Participants represented a diverse mix of roles: 54% were researchers, 40% were patients, 29% were healthcare providers, and 14% were policymakers. Notably, several attendees self-identified with more than one role (e.g., healthcare provider and researcher, or patient and researcher). This overlap was considered a strength of the event, as it fostered interdisciplinary exchange and reflection across professional and experiential perspectives. Consequently, role-based percentages are not mutually exclusive and exceed 100%. Participants were drawn from multiple European countries, including Italy, Croatia, Poland, Romania, Latvia, Portugal, Slovenia, Ireland, and Malta, providing a broad international perspective that enriched discussions and comparative insights across healthcare systems.

Participants’ familiarity with digital health technologies varied (*M* = 2.24, *SD* = 0.68). Almost 14% (13.8%) of participants reported being *not very familiar*, indicating limited experience with digital health tools. Nearly half (48.2%) were *somewhat familiar*, indicating moderate experience and comfort with basic functions but not with advanced features. The remaining participants (38%) were *very familiar*, representing confident users who regularly engage in personal or professional contexts.

Participants also reported frequent use of digital technologies for health-related purposes (*M* = 3.00, *SD* = 0.95). Specifically, 38.2% indicated using such technologies *weekly* and 35.3% *daily*, whereas only 17.6% reported *monthly* use and 8.8% reported *rare* use, suggesting a generally high level of engagement with digital health resources among respondents.

### System usability scale

Overall, the Beacon DSS received a mean SUS score of 60.3 (*SD* = 9.6), which falls within the range typically interpreted as “acceptable but in need of improvement.” While this score suggests that users were generally able to operate the system and complete basic tasks, it also reflects lingering usability challenges, particularly in navigation efficiency, consistency, and intuitiveness. The standard deviation indicates some variability in experiences, likely reflecting differences in users’ technological familiarity, expectations, and professional roles. Table 2 presents the item-level SUS results. As the SUS includes both positively and negatively worded items, higher scores indicate more favorable usability for positively phrased items, whereas lower scores are desirable for negatively phrased items (e.g., perceived complexity or need for technical support).

**Table 2:** Item-level results of the System Usability Scale (SUS)

Question	Mean	SD
I think I would like to use this system frequently	3.82	0.54
I found the system unnecessarily complex	2.01	0.95
I thought the system was easy to use	4.01	0.62
I think I would need the support of a technical person to be able to use this system	1.96	1.20
I found that the various functions of this system were well integrated	3.17	0.81
I thought there was too much inconsistency in the system	2.57	0.63
I imagine that most people would learn to use this system very quickly	3.82	0.61
I found the system very cumbersome to use	2.53	0.57
I felt very confident using the system	3.82	0.47
I needed to learn many things before I could start using this system	1.71	0.85

### AttrakDiff mini scores

**Fig. 4:**
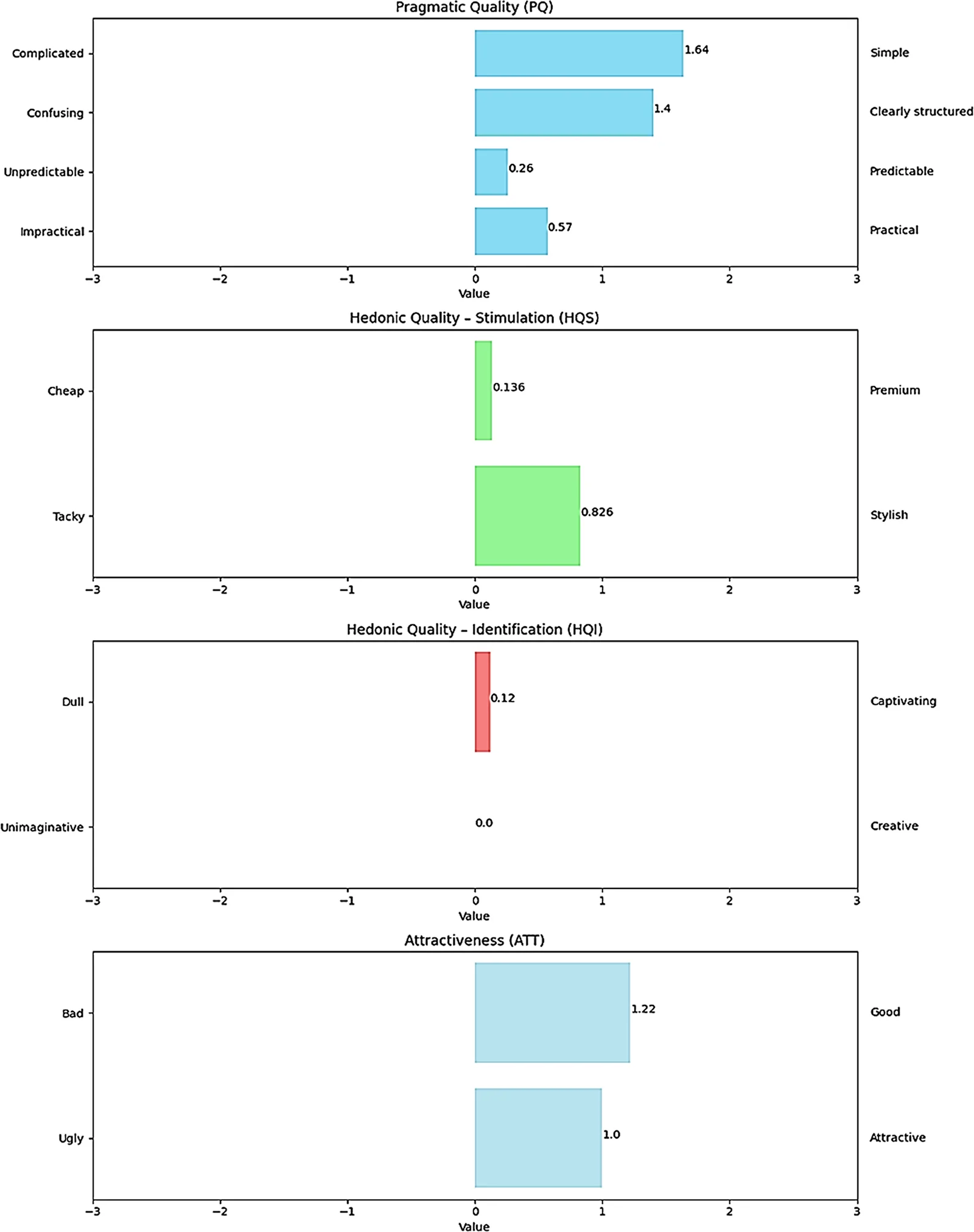
AttrakDiff mini results for the Beacon decision support system. The graph displays mean user ratings across four dimensions of user experience: PQ, HQS, HQI, and ATT. Scores range from − 3 (very negative) to + 3 (very positive), with higher values indicating more favorable perceptions of usability and emotional engagement

To better understand how users experienced the Beacon DSS functionalities, as well as the emotional and aesthetic aspects, we analyzed responses using the AttrakDiff Mini, which evaluates four core dimensions of user experience: Pragmatic Quality (PQ), Hedonic Quality – Stimulation (HQS), Hedonic Quality – Identification (HQI), and Attractiveness (ATT). As shown in Fig. 4, the results revealed a positive rating for Pragmatic Quality, indicating that users found the app practical, easy to navigate, and well-structured (Simple: *M* = 1.64 *SD* = 1.60); Clearly structured: *M* = 1.40 *SD* = 1.14; Predictable: *M* = 0.26 *SD* = 1.02; Practical: *M* = 0.57 *SD* = 1.24). The interface was perceived as predictable and simple, supporting its utility in guiding users through complex oncology information. These findings affirm that the Beacon DSS fulfills core functional expectations, a critical foundation for digital health tools intended for broad, cross-stakeholder use. In terms of Hedonic Quality – Stimulation (HQS), responses were moderately positive (Premium: *M* = 0.14 *SD* = 1.14; Stylish: *M* = 0.83 *SD* = 1.18). Users appreciated the visual clarity and clean design but felt the app lacked novelty or emotional engagement. While the interface was described as pleasant and minimally distracting, some participants suggested that additional visual richness, personalization, or interactive elements could elevate the experience. For Hedonic Quality – Identification (HQI), scores were neutral (Captivating: *M* = 0.12 *SD* = 1.16; Creative: *M* = 0.00; *SD* = 0.80), indicating that while users did not feel alienated by the design or content, the app did not yet foster a strong sense of personal connection or alignment with users’ values. This suggests that further attention to tone, language, and role-based customization (e.g., patient vs. clinician interface views) may enhance emotional resonance. Lastly, Attractiveness (ATT) was positive (Good: *M* = 1.22 *SD* = 0.78; Attractive: *M* = 1.00 *SD* = 0.71), reflecting a favorable general impression among users. The DSS was considered visually appealing and credible, suggesting that, despite some functional and hedonic limitations, it is well positioned to gain user trust and sustain engagement.

Finally, when asked to rate their experience using a single-item global satisfaction score, participants reported a mean satisfaction level of 3.25 out of 5 (*SD* = 0.54). This rating reflects moderate satisfaction, with relatively low response variability, indicating a consistent impression across the sample. While users appreciated the concept, structure, and content of the app, the score also underscores a shared desire for enhancements - particularly in interactivity, speed, personalization, and guided onboarding.

## Discussion

Human-Computer Interaction (HCI) plays a crucial role in the design and success of DSS in healthcare [24, 25, 36]. While DSS aims to provide clinicians, patients, and policymakers with timely and relevant information for decision-making, the effectiveness of these systems depends heavily on how users perceive, navigate, and trust the technology [22, 23]. HCI principles ensure that DSS are not only functional and accurate but also user-friendly, intuitive, and responsive to users’ cognitive and emotional needs - particularly important in high-stakes, sensitive domains like healthcare [24, 25]. By integrating HCI approaches, developers can create DSS that enhance usability, foster user engagement, and ultimately support better health outcomes [24, 25].

This study evaluated the usability and acceptability of the Beacon DSS. Findings show that the platform is well-received for its visual design, content credibility, and simple structure. However, critical gaps in search logic, language settings, personalization, and data completeness still limit its potential impact. The qualitative findings revealed that while users appreciated the Beacon DSS’s clear design, visual accessibility, and credible content, several challenges limited its usability. Both patients and professionals reported technical issues, including freezing and slow loading. The lack of advanced filters and the overwhelming number of search results made navigation difficult, especially when seeking rare cancer services or clinical trials. Emotional aspects also emerged, with patients expressing discomfort with medical terminology and requesting more empathetic, patient-friendly language. Effective, clear, and compassionate communication improves patient-centered outcomes, especially among vulnerable groups [37]. Although this is well established in doctor–patient relationships, growing evidence indicates that similar communication qualities are crucial for digital tools such as AI systems, chatbots, and decision support platforms [37–40]. Whether from healthcare professionals or digital technologies, clear and patient-centered communication fosters trust, reduces anxiety, and supports well-being and informed decision-making, all of which significantly affect healthcare outcomes [39–42]. Participants also suggested improvements, including clearer homepage organization, integrated tutorials, a glossary, and AI-based support. These insights emphasize the need for the DSS to be not only functional but also emotionally sensitive, especially in the context of oncology care [43].

On the other hand, the SUS score suggests that while the system offers basic functionality, there is room for refinement, particularly for users with lower digital proficiency. Overall satisfaction appears moderate. The AttrakDiff results indicate that the system meets functional needs (PQ) and is broadly attractive (ATT), with HQS and HQI pointing to opportunities to enhance further stimulation and personalization, rather than indicating any major shortcomings. Taken together, these findings suggest that while the system establishes a good foundation in usability and appeal, its long-term success may depend on improving the user experience through more engaging, intuitive, and tailored interactions, especially to better support a wider range of users, including those less familiar with digital technologies.

### Clinical implications and future directions

The findings of this evaluation have important implications for the future development and integration of digital support systems in oncology care. As cancer patients increasingly turn to digital tools to navigate their care journeys, platforms like the Beacon DSS can play a critical role in addressing information gaps, facilitating access to specialized services, and empowering users to make decisions [4, 14, 15]. Importantly, the DSS may also contribute to improved accessibility and potentially support a more equitable access to cancer care. Features such as searchable hospital directories, treatment option filtering, and accessible content can empower patients who may otherwise lack information or social support. In addition, multilingual support, transparent information on oncology services, and centralized navigation across healthcare resources may help reduce informational and navigational barriers. Healthcare professionals also noted that the platform can help identify gaps in local service provision, support referrals, and potentially inform resource allocation in underrepresented areas. Taken together, these features represent plausible mechanisms through which the Beacon DSS may enhance accessibility, transparency, and informed decision-making, particularly for diverse patient populations, thereby addressing a key research gap highlighted in the introduction. Nevertheless, evaluating whether these mechanisms translate into reduced disparities in access to cancer care was beyond the scope of the present usability-focused study.

Future efforts to support accessibility and equity through digital oncology tools should also consider the broader user experience, including emotional, linguistic, and personalization needs. To fully realize this potential, usability must be paired with emotional sensitivity, inclusivity, and personalization. Users’ requests to soften terminology and simplify language, particularly for patients experiencing psychological distress, highlight the need for tools that go beyond functional design. Emotional usability, including the tone, vocabulary, and clarity of content, should be a central consideration in digital oncology, especially for populations who may experience heightened vulnerability due to diagnosis-related anxiety, cognitive fatigue, or comorbid psychiatric or neurological conditions [44, 45]. At the same time, preferences regarding emotionally sensitive terminology are likely to vary across individuals and contexts, and future research should explore how empathy-oriented language can be balanced with clarity, accuracy, and accessibility in digital oncology tools. In addition, the underrepresentation of smaller cancer centers and the limited depth of coverage of rare cancers or psychosocial services suggest that data inclusivity is a key priority. Enhancing the DSS’s database through structured partnerships with local hospitals, patient associations, and care providers could not only improve content accuracy but also strengthen its perceived utility across a broader spectrum of users [46, 47]. From a clinical workflow perspective, study stakeholders appreciated the platform’s credibility and referral value. They also emphasized the need to enhance the DSS to make it a seamless extension of clinical practice, including time-efficient searches, patient education, and informed referrals.

Future development could also consider incorporating adaptive user profiles and AI-driven assistance to tailor the experience to user type, literacy level, or language preference. These features may improve both engagement and usability, particularly among users with lower digital confidence or limited exposure to health technologies. Finally, ongoing usability testing should be embedded into the platform’s development cycle. As new features are introduced and as user expectations evolve, continuous, iterative evaluation will be critical to ensure that the Beacon DSS remains both relevant and responsive to the needs of its diverse audience.

### Limits and strengths of the study

This study provides valuable insights into digital health and cancer care, as it is the first formal evaluation of the Beacon DSS. Using a mixed-methods approach, it combined in-depth qualitative feedback from key stakeholders with quantitative usability metrics to capture both technical and emotional factors influencing engagement. A notable strength is the platform’s responsive development: several user-reported issues, such as slowness and freezing, were promptly addressed, reflecting a user-centered and flexible approach. The DSS shows promise as a decision-support tool for multiple stakeholders, centralizing access to validated oncology resources and referral networks to support more informed and equitable care decisions. Limitations include the modest sample size and participants’ generally medium-to-high digital literacy, which may limit generalizability to broader or less tech-savvy populations. Furthermore, the sample may underrepresent individuals with specific accessibility needs related to disability or cognitive burden. In some cases, users with severe or complex disabilities may interact with the system through caregiver-mediated access. In addition, although the platform supports several European languages, the evaluation did not specifically assess differences in usability across all language groups. Nevertheless, consistent themes across qualitative and quantitative data highlight key usability priorities. For this reason, we plan to replicate and expand these findings across larger, more diverse populations and incorporate longitudinal or cross-country analyses to assess the tool’s broader impact on oncology care and decision-making. Another limitation is that although the Beacon DSS was designed to support more equitable access to oncology information and services, the present evaluation focused exclusively on usability and user experience. Therefore, no conclusions can be drawn regarding the platform’s impact on healthcare disparities, equitable access to cancer care, or related clinical outcomes. Finally, subgroup analyses by stakeholder role were not feasible for the quantitative evaluation as responses were collected anonymously, during a live workshop, and using an audience-response platform. Consequently, the quantitative findings reflect overall perceptions of usability rather than stakeholder-specific experiences. Although the Beacon DSS is intended for multiple stakeholder groups, the present findings should be interpreted in light of the stakeholder representation achieved in this evaluation, with further studies warranted to more fully assess the perspectives of underrepresented groups, including policymakers. 

## Conclusion

Overall, the integration of qualitative and quantitative findings provided a preliminary complementary understanding of the Beacon DSS user experience. While qualitative data offered insights into usability challenges and user expectations, quantitative results confirmed the platform’s acceptable usability and helped contextualize areas for further improvement. Iterative development based on user feedback - especially enhancements to filtering, onboarding, emotional tone, and database depth - will be critical to ensure adoption across patient and professional communities. As digital navigation tools become central to patient empowerment in complex care systems, continuous user-centered evaluation must remain a cornerstone of their evolution.

## Supplementary Information

Below is the link to the electronic supplementary material.

Supplementary Material 1

## Data Availability

Data is available from the corresponding author on reasonable request.

## Declarations

### AI-use declaration

During the preparation of this manuscript, the authors used an AI-based language model (ChatGPT, OpenAI) solely for English language editing. The authors subsequently reviewed, revised, and approved the final content and take full responsibility for the manuscript.
