# Supplementary material for: Evaluating the user experience of the Beacon Decision Support System: a mixed-methods study of a digital platform for equity-oriented cancer care
Source: BMC Health Serv Res. 2026 Jul 27;26:1459. doi: 10.1186/s12913-026-15210-w (PMC13692986; doi:10.1186/s12913-026-15210-w)
Supplement: Supplementary file 1 [file 12913_2026_15210_MOESM1_ESM.docx]

**Supplementary File 1**

Supplementary Table 1 (S1): Summary of stakeholders, corresponding functionalities, expected benefits, and illustrative use cases of the Beacon DSS

| **Stakeholder** | **Functionality** | **Benefit** | **Example Use Cases** |
| --- | --- | --- | --- |
| *Patients* | Multilingual interface available in 24 languages, patient-oriented navigation (including adapted layouts and color schemes), and search filters by geographical area and cancer type. Access to validated directories of accredited hospitals, clinical trial registries, patient organizations, and educational resources. | Simplifies access to reliable information by bringing together trusted resources within a single platform. Reduces language and navigation barriers across European healthcare systems and supports informed healthcare decisions, promoting more equitable access to specialized services. | Identifying the closest OECI-accredited center with expertise in a rare cancer; searching for relevant clinical trials locally or across Europe; finding patient associations and support organizations to facilitate psychosocial support. |
| *Healthcare Professionals* | Dedicated professional interface with advanced search functions for diagnostic and therapeutic services, providing centralized access to information from multiple institutions. Includes evidence-based clinical guidelines, European oncology networks, centers of excellence, training opportunities, and institutional capability profiles. | Streamlines clinical decision-making by reducing the need to consult multiple external resources. Encourages collaboration among institutions and supports evidence-informed referrals, particularly for patients requiring highly specialized care. | Referring a patient to a specialist oncology center; identifying suitable clinical trials during a consultation; verifying the availability of specific diagnostic or therapeutic services at neighboring institutions. |
| *Researchers* | Harmonized datasets integrating information from hospitals, cancer registries, administrative claims databases, biobanks, and other research resources. Data are organized using Resource Description Framework (RDF) and Dublin Core standards, enabling advanced SPARQL queries and large-scale analytical approaches. | Promotes interoperability across heterogeneous European data sources, facilitates multinational research collaborations, and enables the identification of geographical and demographic gaps in cancer research and healthcare delivery. | Querying centers with specific diagnostic capabilities and associated biobanks to support multicenter studies; analyzing registry and claims data to investigate regional differences in cancer outcomes; mapping specialized healthcare resources to identify potential sites for future clinical trials. |
| *Policymakers* | Decision-support tools including the Cancer Disparity Atlas, NLP-derived policy analyses, aggregated registry and claims data, and visual summaries of healthcare infrastructure across Europe. | Provides objective, population-level evidence to identify disparities in cancer care, resource allocation, and healthcare infrastructure, thereby supporting evidence-based policy development and strategic planning. | Identifying underserved regions with high cancer mortality and limited access to accredited oncology centers to guide investment decisions; evaluating the impact of cancer policies using aggregated healthcare data; assessing geographical variation in the implementation of evidence-based cancer care across European countries. |
